# Exploration of the Key Proteins in the Normal-Adenoma-Carcinoma Sequence of Colorectal Cancer Evolution Using In-Depth Quantitative Proteomics

**DOI:** 10.1155/2021/5570058

**Published:** 2021-06-11

**Authors:** Yin Zhang, Chun-Yuan Li, Wei Ge, Yi Xiao

**Affiliations:** ^1^Division of Colorectal Surgery, Department of General Surgery, Peking Union Medical College Hospital, Chinese Academy of Medical Sciences and Peking Union Medical College, Beijing, China; ^2^State Key Laboratory of Medical Molecular Biology and Department of Immunology, Institute of Basic Medical Sciences Chinese Academy of Medical Sciences, School of Basic Medicine Peking Union Medical College, Beijing, China

## Abstract

**Purpose:**

In most cases, the carcinogenesis of colorectal cancer (CRC) follows the normal-adenoma-carcinoma (N-A-C) sequence. In this study, we aimed to identify the key proteins in the N-A-C sequence.

**Methods:**

Differentially expressed proteins (DEPs) in normal, adenoma, and carcinoma tissues were identified using the Tandem Mass Tag- (TMT-) based quantitative proteomics approach. The landscape of proteomic variation in the N-A-C sequence was explored using gene set enrichment analysis (GSEA) and Proteomaps. Key proteins in the N-A-C sequence were identified, verified, and validated based on our proteomic data, external proteomic data, and external transcriptomic data in the ProteomeXchange, CPTAC, GEO, and TCGA databases. The prognostic value of the key proteins in our database was evaluated by univariate and multivariate Cox regression analysis. The effects of the key proteins on adenoma organoids and colorectal cancer cells were explored in functional studies.

**Results:**

Based on our proteomic profiles, we identified 1,294 DEPs between the carcinoma (CG) and normal (NG) groups, 919 DEPs between the adenoma group (AG) and NG, and 1,030 DEPs between the CG and AG. Ribosome- and spliceosome-related pathways were mainly enriched in the N-A process. Extracellular matrix- and epithelial-mesenchymal transition- (EMT-) related pathways were mainly enriched in the A-C process. RRP12 and SERPINH1 were identified, verified, and validated as candidate key proteins in the N-A and A-C processes, respectively. Furthermore, RRP12 and SERPINH1 knockdown impeded the viability and proliferation of adenoma organoids. SERPINH1 was validated as a risk factor for disease-free survival (DFS) based on the TCGA and our database, whereas RRP12 did not show prognostic value. SERPINH1 knockdown was accompanied by EMT-related protein variation, increased apoptosis, and reduced proliferation, invasion, and migration of CRC cells in vitro.

**Conclusions:**

RRP12 and SERPINH1 may play an important role in the N-A and A-C processes, respectively. Furthermore, SERPINH1 showed favorable prognostic value for DFS in CRC patients. We speculate that SERPINH1 might promote not only the A-C process but also the development of CRC.

## 1. Introduction

Colorectal cancer (CRC) is the second leading cause of cancer deaths, accounting for approximately 9.2% of all cancer deaths annually [1]. Approximately 85% of CRC cases evolve following the normal-adenoma-carcinoma (N-A-C) sequence, which is based on the accumulation of genetic mutations and chromosomal instability (CIN) [2]. The canonical model of the colorectal carcinogenesis requires chronological gene mutations. Inactivation of the adenomatous polyposis coli (APC) initiates the progression from normal mucosa to adenoma, and then Kras and TP53 mutation occurs followed by multiple additional gene mutations [3]. The majority of the subsequent mutations cannot be accounted for by the fundamental rate of genetic changes but require the development of intrinsic genomic instability for the new mutations to arise [4]. Hence, CIN is the driver of tumorigenesis and the loss of heterozygosity at chromosome 18q is one of the most frequent CIN events [5].

Based on the major principle of carcinogenesis, the majority of studies on colorectal cancer have employed a genomic analysis approach to explore the N-A-C sequence [6–11]. Some researchers attempted to identify the driver events of this sequence at the level of transcriptomics by investigating original or public RNA sequencing data [12, 13]. From the clinical perspective, adenoma is a representative premalignant lesion and should be removed during colonoscopy. Alternatively, if direct resection is difficult or hazardous, colectomy should be performed. Old age, family history of cancer, smoking, nonuse of nonsteroidal anti-inflammatory drugs (NSAIDs), and a large volume adenoma can increase the risk of adenoma carcinogenesis [2]. The course of carcinogenesis from normal mucosa to carcinoma is up to 15 years. Only 2.5% to 5.6% of cases progress to advanced adenoma (≥1 cm in size, tubulovillous or villous adenoma, high grade dysplasia), which is associated with a high risk of malignant transformation [14, 15].

The low rate of carcinogenesis from adenoma indicates that the driver events of the process of transformation from normal mucosa to adenoma (N-A) may be different from that from adenoma to carcinoma (A-C). Furthermore, in the N-A-C sequence, the “mutational hits” in the N-A process are different from those in the A-C process [5]. Hence, the canonical N-A-C evolution of colorectal cancer requires focused investigations that are distinct from those focusing on the separate N-A and A-C processes. In this study, we aimed to identify key proteins which may possess diagnostic and prognostic value in the N-A-C sequence in the evolution of CRC.

## 2. Methods

### 2.1. Patients and Tissue Samples

Normal, adenoma, and carcinoma tissues were obtained from 23 patients recruited in the Division of Colorectal Surgery, Department of General Surgery, Peking Union Medical College Hospital (China). The clinical and pathological characteristics of the patients are listed in Supplementary Table 1. All of the tissues were stored temporarily on the dry ice after collection and then transferred to −80°C prior to analysis. This study was approved by the Ethics Committee of Peking Union Medical College Hospital (number: JS-2094) and complied with the Declaration of Helsinki. Written informed consent was obtained from each patient prior to study commencement.

### 2.2. Tandem Mass Tag-Labeling (TMT) and Mass Spectrometry Data Analysis

Lysate proteins (100 *µ*g) in 8 M urea were reductive with dithiothreitol (DTT) and were alkylated with iodoacetamide (IAA). Then the proteins were dissociated with Trypsin/Lys-C, and peptide labeling was performed using the TMT Kit. The TMT-labeled peptides were subjected to high-performance liquid chromatography (HPLC) analysis followed by analysis with a directly interfaced Thermo Orbitrap Fusion mass spectrometer (Thermo Scientific). Protein identification was performed using Proteome Discoverer 2.2 software (Thermo Scientific) with the SEQUEST search engine. Only proteins with a false discovery rate (FDR) < 0.01 and unique peptide ≥2 qualified for the further analysis. Relative protein quantification was performed using the TMT-6plex method. All methods were performed as previously described [16]. The mass spectrometry proteomics data have been deposited to the ProteomeXchange Consortium (http://proteomecentral.proteomexchange.org) via the iProX partner repository with the dataset identifier: PXD017068 and PXD023899 [17].

### 2.3. Transcriptomic Data Collection

The gene expression profiles and corresponding clinical information were acquired from the GEO database (https://www.ncbi.nlm.nih.gov/geo/). The gene expression matrices of the GSE 117606, GSE 50014, and GSE 50015 datasets were downloaded for coanalysis with the proteomic data. Differentially expressed genes (DEGs) between normal tissues and adenoma tissues and between adenoma tissues and carcinoma tissues in the three datasets were identified using the GEO2R tool (https://www.ncbi.nlm.nih.gov/geo/geo2r/) following the criteria of adjusted *P* value < 0.05 and |log_2_ fold change (FC) ratio| >0.3. GSE 20916 and GSE 41567 datasets were used to validate the key proteins.

### 2.4. Immunohistochemistry (IHC)

Paraffin-embedded normal, adenoma, and carcinoma tissue samples were cut into 4 *µ*m thick sections. Tissue sections were deparaffinized in xylene, hydrated in ethanol, and boiled with citrate buffer for antigenic retrieval following standard IHC procedures. The sections were then incubated with anti-SERPINH1 (1 : 500 dilution, Sigma) and anti-RRP12 (1 : 200 dilution, Thermo) for 24 h. On the second day, the sections were incubated with second antibodies (Zhongshan Biotech, Beijing, China) and stained with DAB (Zhongshan Biotech, Beijing, China). IHC scores were calculated as the product of stain-positive cell score and the staining intensity score. The stain-positive cell score was determined as follows: 1 = <25% cells, 2 = 25%–50% cells, 3 = 50%–75% cells, and 4 = >75% cells. Staining intensity was determined as follows: 0 = no staining, 1 = low-level staining, 2 = intermediate-level staining, and 3 = high-level staining. High and low IHC scores were defined as 9–12 and 0–8, respectively.

### 2.5. Bioinformatic Analysis

Common proteins in the three replicates were identified using a Venn diagram webtool (http://jvenn.toulouse.inra.fr/app/example.html) [18]. Hierarchical clustering analysis was performed using R 3.6.1 (https://cran.r-project.org/bin/windows/base/) and the “Pheatmap” package. Gene set enrichment analysis (GSEA) software and the molecular signature database, MsigDB, were downloaded from the Broad Institute website (http://www.broadinstitute.org/gsea/index.jsp) [19]. The canonical pathways (CP) and gene ontology gene sets were used to analyze the differences in the proteomic profiles between the normal group (NG) and adenoma group (AG) and between the AG and carcinoma group (CG). Proteomaps (http://www.Proteomaps.net/) were used to show the function of the differentially expressed proteins (DEPs) using the polygon module based on the Kyoto Encyclopedia of Genes and Genomes (KEGG) pathways gene classification [20]. The size of each polygon module represents the relative abundance of the enriched proteins. The protein-protein associations of the intersected genes were identified using Search Tool for the Retrieval of Interacting Genes (STRING) 11.0 (https://string-db.org/) [21]. We then identified the candidate key genes based on the “MCODE” and “cytoHubba” plugins in Cytoscape 3.7.0 [22, 23]. The ranking of the candidate key genes was visualized using the heatmap application of TB tools [24]. GEPIA (http://gepia.cancer-pku.cn/) and UALCAN are online databases including colorectal normal and cancer gene expression data, corresponding with the clinicopathological and prognostic information of the Cancer Genome Atlas (TCGA) [25, 26]. The hub genes were validated by reference to the external proteomic data of clinical proteomic tumor analysis consortium (CPTAC), which are standardized and shown in UALCAN.

### 2.6. Establishment of Human Adenoma Organoids

Fresh adenoma tissue was cut into small pieces, washed with PBS, and dissociated in TrypLE (Gibco, 12604-013) to achieve cell suspension. We then used Sato's method to prepare the culture medium, which was changed every 3 days [27]. The organoids were passaged (1 : 3) every week. Appropriate Cell Recovery Solution (Corning, REF354253) was added to the Matrigel and dissociated for 1 h at 4°C to obtain the organoid suspension. Then the supernatant was discarded after centrifugation, and the organoids were embedded in Matrigel.

### 2.7. Cells siRNA Transfection

SW480 cells were seeded 1 day in advance to ensure that the cell confluence reached 70%–80% before transfection. According to the instructions, Opti-MEM (Gibco, 31985-062, USA) was used to dilute RNAiMAX (Invitrogen, 13778-150, USA), SERPINH1-siRNA1, siRNA2, and siRNA3 (RiboBio, China), respectively. The diluted RNAiMAX was mixed with each siRNA dilution and the mixed dilution was added to the cell culture medium after standing still for 5 min. The final concentration of SERPINH1-siRNA1, siRNA2, and siRNA3 was 50 nM, respectively. After incubating at 37°C for 48 hours, the cells were harvested for subsequent experiments.

### 2.8. Adenoma Organoids siRNA Transfection

Cell Recovery Solution (Corning, REF354230) was used to dissociate Matrigel. Organoids pellet was obtained after centrifugation, and then the centrifugation was dissociated by TrypLE (Gibco, 12604-013) for 10 min at 37°C. SERPINH1-siRNA2 was diluted according to the above description and the final concentration was 50 nM. After centrifugation, the organoids were incubated at 37°C for 4 hours. The organoids were collected and seeded in Matrigel for further incubation after removing the supernatant. After 48 hours, they were collected for subsequent experiments.

### 2.9. Immunofluorescence Staining

After transfection with siRNAs, organoids were fixed with 4% paraformaldehyde and blocked. Organoids were then incubated with anti-Ki-67 (Abcam, ab15580, Rabbit, 1 : 100) at 4°C overnight. Then, cells were incubated with Alexa Fluor 488 conjugated Goat anti-Rabbit IgG (Life Technologies, A11034, 1 : 100) at 4°C overnight. Finally, the pictures were captured using confocal microscope (ZEISS LSM 780, Germany).

### 2.10. RNA Isolation, qRT-PCR, and Droplet Digital PCR (ddPCR) Analysis

Total RNA was extracted from the SW480 cell lines using TRIzol reagent (Thermo Scientific) according to the manufacturer's instructions. RNA was reversely transcribed into cDNA using the QuantiTect Reverse Transcription Kit (QIAGEN, Valencia, CA, USA) according to the manufacturer's instructions. Real-time quantitative PCR was performed using SYBR-Green (Takara, Otsu, Shiga, Japan), and the expression levels were normalized to that of GAPDH. The quantity of DNA in adenoma organoids was evaluated by ddPCR. Approximately 200 ng DNA extracted from each organoids was analyzed in 14.5 *μ*l of the ddPCR reaction mixture.

The qRT-PCR was performed using the following primers:

  GAPDH (forward: 5′-GTCTCCTCTGACTTCAACAGCG-3′; reverse: 5′-ACCACCCTG-TTGCTGTAGCCAA-3′)
  RRP12 (forward: 5′-GTGACCTGACAGTCGATGCTG-3′; reverse: 5′-GTGACGTTTGTGCAGTCGG-3′)
  SERPINH1 (forward: 5′-TCAGTGAGCTTCGCTGATGAC-3′; reverse: 5′-CATGGCGTTGACTAGCAGGG-3′)

### 2.11. CCK8 Assay

SW480 cells were seeded in 96-well plates (3,000 cells/well). The proliferation ability of cells was evaluated using the Cell Counting Kit-8 (MCE, HY-K0301) according to the manufacturer's protocols. After culturing for 2–3 h, the absorbance in each well was measured at a wavelength of 450 nm using a multimicroplate test system.

### 2.12. 3D Cell Viability Assay

The adenoma organoids transfected with SERPINH1-siRNA (refer to the method: adenoma organoids siRNA transfection) was used to detect cell viability. The CellTiter-Glo^®^ 3D Reagent (Promega, G9681) was used to dissolve Matrigel. The adenoma organoids suspension was acquired and then was equally distributed to opaque-walled multiwell plates (Corning, REF3603). A volume of CellTiter-Glo^®^ 3D Reagent equal to the volume of cell culture medium was added to each well and then the content was incubated for 30 min at room temperature. Finally, total ATP content of each well determines the cell viability.

### 2.13. Apoptosis Analyses

For cell apoptosis analysis, cells were incubated for 48 h following transfection and then processed with a FITC Annexin-V Apoptosis Detection Kit (Invitrogen, USA) according to the manufacturer's instructions and analyzed by flow cytometry. Data were analyzed using FlowJo 10.7.1 software.

### 2.14. Transwell Migration and Invasion Assays

Cell invasion and migration abilities were evaluated using Transwell assays. For the invasion assay, Matrigel chambers (Corning, NY, USA) were used according to the manufacturer's instructions. A total of 2.5 × 10^4^ cells/well were resuspended in 100 *μ*l fetal bovine serum-free medium in the upper chamber (8 *μ*m pore size, CoStar, Corning, NY, USA) of a Transwell system. The lower chamber was filled with 0.6 ml medium supplemented with 10% FBS. After incubation for 48 h at 37°C, the invasive cells were fixed with 100% methanol and stained with 0.5% crystal violet before counting under an inverted microscope. For the migration assay, 2.5 × 10^4^ cells were plated in uncoated Transwell upper chambers. The number of cells that migrated across the membrane was estimated under an inverted microscope (Nikon, Tokyo, Japan) at 20× magnification.

### 2.15. Western Blot Analysis

Protein concentration was determined using BCA Protein Assay Kits (Thermo Scientific, USA) according to the manufacturer's instructions. All protein samples were separated by 10% sodium dodecyl sulfate-polyacrylamide gel electrophoresis, transferred to a polyvinylidene fluoride membrane, and incubated overnight at 4°C with primary antibodies for the detection of the following: SERPINH1 (Sigma, #386023), *β*-catenin (CST, #8480), NF-*κ*B p65 (phospho-S536, Abcam, ab76302), Twist (Abcam, ab50887), Slug (Abcam, ab27568), GSK-3*β* (CST, #12456), phospho-GSK-3*β* (Ser9, CST, #5558), N-cadherin (CST, #13116), E-cadherin (CST, #8193), MMP9 (Abcam, ab137867), and MMP14 (Abcam, ab51704). The membrane was then washed and incubated with a horseradish peroxidase-conjugated secondary antibody.

### 2.16. Statistical Analysis

Statistical analysis was performed using GraphPad Prism 8.0.1 (GraphPad Software, Inc., La Jolla, CA, USA). The significance of DEPs (*P* < 0.05 and |log_2_ FC ratio| >0.3) identified in paired comparisons of NG, AG, and CG was evaluated using Student's *t*-test based on the grouped proteomic abundance and depicted volcano plot. Receiver operating characteristic (ROC) curves were generated to validate the discrimination value of RRP12 and SERPINH1. The Kaplan–Meier method was used for survival analysis. Univariate and multivariate Cox regression analyses were used to identify independent prognostic factors. The criterion for inclusion in the multivariate Cox regression analysis was *P* < 0.1. *P* < 0.05 was considered to indicate statistical significance.

## 3. Results

### 3.1. Proteomic Profiles of Normal, Adenoma, and Carcinoma Tissues

**Figure 1:**
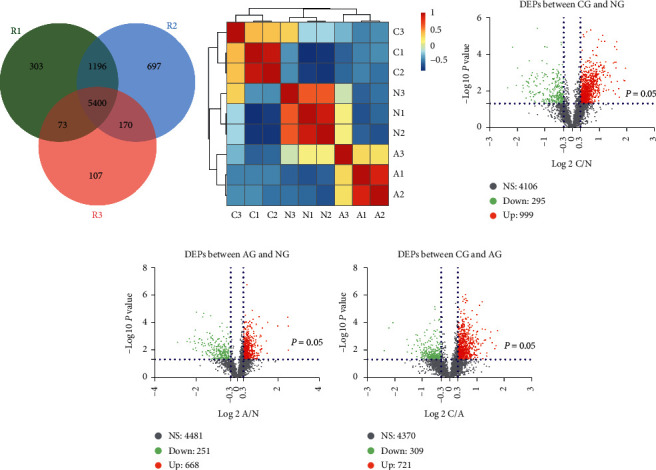
Proteomic profiles of normal mucosa, adenoma, and carcinoma tissues. (a) Venn diagram showing the intersection of the available proteins identified in the three duplicates. (b) Hierarchical clustering analysis of the grouped proteomic profiles of normal mucosa, adenoma, and carcinoma tissues in each replicate. C1: proteomic profiles of carcinoma in R1; C2: proteomic profiles of carcinoma in R2, and so forth. (c) Volcano plot of the DEPs between CG and NG. (d) Volcano plot of the DEPs between AG and NG. (e) Volcano plot of the DEPs between CG and AG. DEPs: differentially expressed proteins; CG: carcinoma group; AG: adenoma group; NG: normal group. NS: no statistically significant differences; Down: downregulated proteins; Up: upregulated proteins.

The workflow of our study is shown in Supplementary Figure 1. A total of 6,972, 7,463, and 5,750 proteins were identified separately in three replicates according the criteria of *q* value <0.01 and unique peptides ≥2 (Supplementary Files 1–3). Keratin proteins were excluded for further analysis because Keratin proteins are mainly expressed in hair and skin and are less likely to be expressed in the colorectal tissues, and Keratin proteins are the main contamination in published contamination list (“contamination.fasta” downloaded from MaxQuant) and are recommended to be excluded [28]. Finally, we identified 5,400 common proteins by integrating the proteomic profiles of the three duplicates (Figure 1(a)). Hierarchical clustering analysis showed a closer correlation degree between NG and AG compared with CG (Figure 1(b)). Comparisons between pairs of groups revealed 1,294 DEPs between CG and NG, 919 DEPs between AG and NG, and 1,030 DEPs between CG and AG (Figures 1(c)–1(e)), *P* value < 0.05 and |log_2_ FC ratio| >0.3). More DEPs were upregulated than downregulated in the N-A, A-C, and N-C processes.

### 3.2. Proteomic Variation Enrichment in the Progression from Normal Mucosa to Carcinoma

It is widely accepted that the N-A and A-C processes represent two phases of colorectal carcinogenesis driven by different events in a sequential manner [5]. Therefore, we explored the proteomic variation enrichment in the two phases separately using the following methods: (1) paired comparison of the proteomic profiles between NA and AG and between AG and CG by GSEA and (2) identification of the biological processes and pathways enriched in the DEPs associated with the N-A and A-C processes.

**Figure 2:**
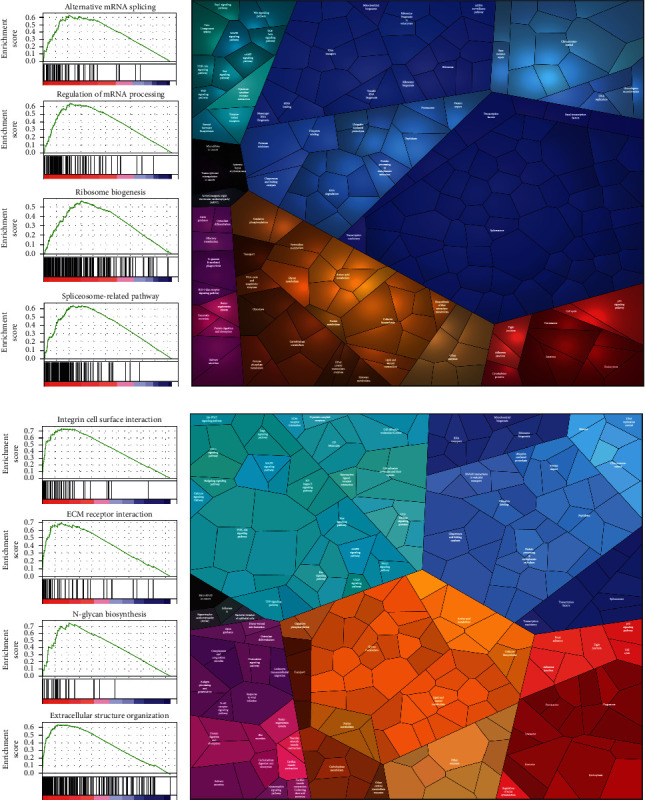
Enrichment analysis of proteomic profiles and DEPs in the N-A and A-C processes. (a) Gene set enrichment analysis (GSEA) showing that the biological process of alternative mRNA splicing (normalized enrichment score (NES) = 2.21, false discovery rate (FDR) < 0.01), regulation of mRNA processing (NES = 2.49, FDR < 0.01), ribosome biogenesis (NES = 2.48, FDR < 0.01), and spliceosome-related pathway (NES = 2.61, FDR < 0.01) were significantly enriched between AG and NG. (b) DEPs between AG and NG were mainly enriched in spliceosome-, RNA transport-, ribosome-, and chromosome-related pathways; the area of each small polygon tile represents a specific protein abundance ratio between AG and NG. (c) GSEA showing that the biological process of extracellular structure organization (NES = 2.97, FDR < 0.01) and the pathway of integrin cell surface interaction (NES = 2.77, FDR < 0.01), extracellular matrix (ECM) receptor interaction (NES = 2.55, FDR < 0.01), and N-glycan biosynthesis (NES = 2.42, FDR < 0.01) were significantly enriched between CG and AG. (d) DEPs between CG and AG were mainly enriched in cancer- and metabolism-related pathways; the area of each small polygon tile represents a specific protein abundance ratio between CG and AG.

In the GSEA, alternative mRNA splicing, regulation of mRNA processing, ribosome biogenesis, and the spliceosome pathway were found to be enriched by comparison of the proteomic profiles of NG and AG (Figure 2(a)). Extracellular structure organization, integrin and cell surface interaction pathway, ECM receptor interaction pathway, and N-glycan biosynthesis pathway were found to be enriched by comparison of the proteomic profiles of AG and CG (Figure 2(c)).

The enrichment analysis of DEPs in the two phases was visualized through the construction of a Proteomap, in which each protein is presented by a small polygon tile. The area of tile reflects the protein abundance ratio of the compared groups. This analysis showed that the DEPs between NG and AG were primarily enriched in spliceosome-, RNA transport-, ribosome-, and chromosome-related pathways (Figure 2(b)). The DEPs between AG and CG were primarily enriched in epithelial-mesenchymal transition- (EMT-) and glycan metabolism-related pathways modules (Figure 2(d)).

The results of the GSEA and Proteomap analysis were similar and both indicated the entirely different progression modes for the N-A and A-C processes.

### 3.3. Integration of Transcriptomic Datasets to Identify and Verify Key Proteins in the N-A-C Sequence

Due to the different proteomic changes of N-A and A-C processes, we separated the two parts and performed integration analysis between the proteomic profiles of each part and the external transcriptomic datasets. Three gene expression profiles (GSE 117606, GSE 50014, and GSE 50015) were downloaded from the GEO database as external transcriptomic datasets to identify upregulated DEGs following the same screen criteria that were applied to our dataset (adjusted *P* value < 0.05 and log_2_ FC ratio >0.3).

**Figure 3:**
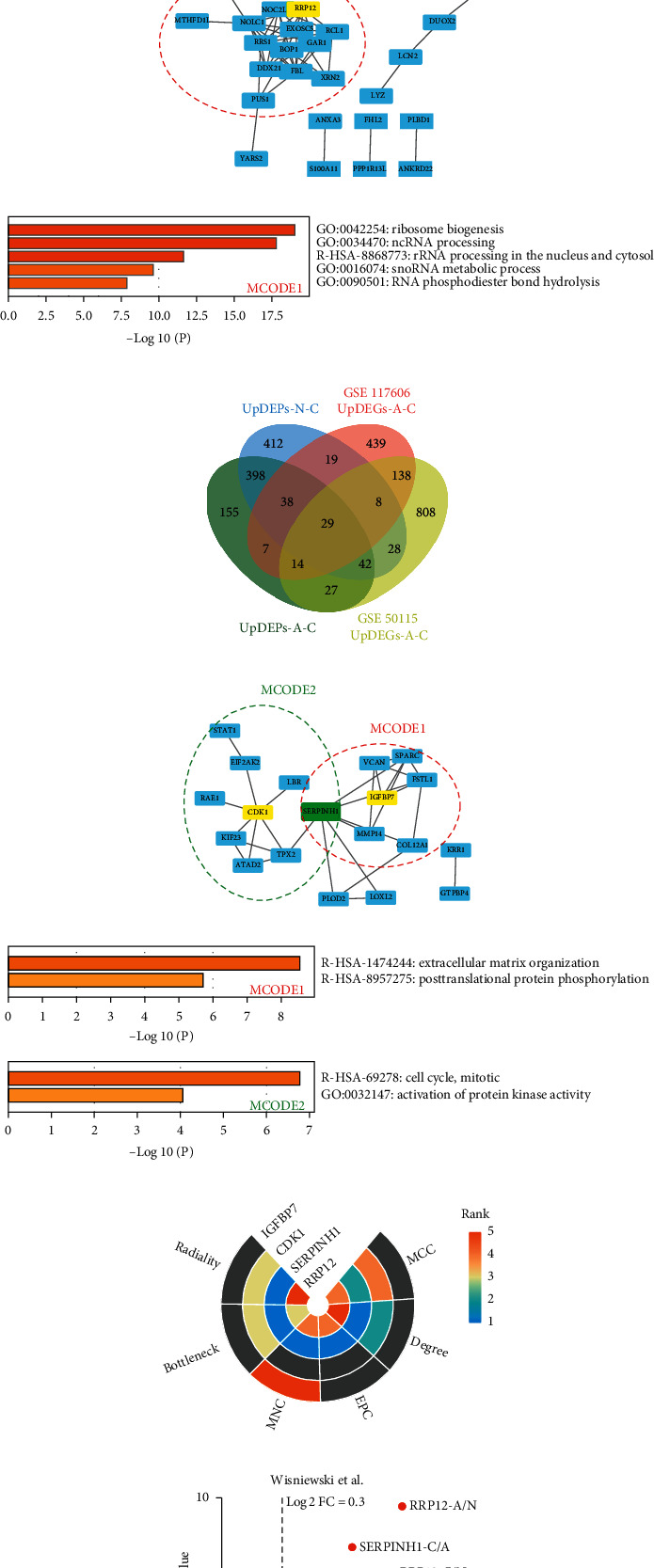
Integrating transcriptomic datasets and proteomic profiles to identify hub proteins in the N-A-C sequence. (a) Venn diagram showing the intersection of upregulated DEPs in the N-C process (UpDEPs-N-C), upregulated DEPs in the N-A process (UpDEPs-N-A), and upregulated DEGs of GSE 117606 and GSE 50114 in the N-A process. A total of 45 intersected genes in the four datasets were identified. (b) The protein-protein interaction (PPI) network of the 45 intersected proteins which could contribute to a connection in the N-A process. The MCODE1 cluster is circled in red and the seed protein (RRP12) is highlighted (MCODE criteria: degree cutoff = 2, node score cutoff ≥ 0.2, K-core ≥ 3, and max. depth from seed = 100; MCODE1 score = 7.231). (c) The primary enrichment of biological processes and pathways in MCODE1 of N-A process. (d) Venn diagram showing the intersection of UpDEPs-N-C, upregulated DEPs in the A-C process (UpDEPs-A-C), and upregulated DEGs of GSE 117606 and GSE 50115 in the A-C process. A total of 29 intersected genes in the four datasets were identified. (e) The protein-protein interaction (PPI) network of the 45 intersected proteins which could contribute to a connection in the A-C process. The MCODE1 and MCODE2 clusters are circled in red and green, respectively. The seed (MCODE1: IGFBP7; MCODE2: CDK1) and hinge protein (SERPINH1) are highlighted (MCODE criteria: degree cutoff = 2, node score cutoff ≥ 0.2, K-core ≥ 3, and max. depth from seed = 100; MCODE1 score = 3.25 and MCODE2 score = 2.222). (f) The primary enrichment of biological processes and pathways in MCODE1 and MCODE2 of the A-C process. (g) Six algorithms (MCC, Degree, EPC, MNC, Bottleneck, and Radiality) were used to analyze and rank the hub proteins of the PPI network in the N-A and A-C processes using the “cytoHubba” plugin. The ranking of RRP12, SERPINH1, CDK1, and IGFBP7 is visualized in a heatmap. Only top five hub proteins in each algorithm are colored as shown in the bar. If the protein was not ranked among the top five, the corresponding column would be gray. Only RRP12 and SERPINH1 were verified among the top five hub proteins using the six algorithms. The rank ranges of RRP12 and SERPINH1 were 3–5 and 1–2, respectively. (h) The variation in the expression of RRP12 and SERPINH1 in the N-A-C sequence was validated using an external dataset reported by Wisniewski et al. based on the criteria of *P* value < 0.05 and log2 FC ratio > 0.3, RRP12 was significantly upregulated in the N-A and N-C processes, and SERPINH1 was significantly upregulated in the A-C and N-C processes. There were no significant differences in the expression of RRP12 in the A-C process and that of SERPINH1 in the N-A process.

In the N-A process, 1,136 upregulated DEGs were identified in GSE 117606 and 3,266 in GSE 50014 by comparison of normal and adenoma tissues. A total of 45 intersected genes were identified in the overlap of upregulated DEGs of the two transcriptomic datasets in the N-A process and DEPs in N-A and N-C processes (Figure 3(a), Supplementary File 4). To further explore the correlation between these proteins and the potential molecular mechanism of their interactions, we constructed PPI networks using STRING v 11.0 (https://string-db.org/, Figure 3(b)). We then used the “MCODE” plugin in Cytoscape to identify core clusters. The MCODE method can be used to identify core clusters that may execute different functions synergistically and contain a seed protein that plays a pivotal role in the cluster. We identified only one cluster using this method and the seed protein, RRP12, was regarded as the candidate key protein in N-A process (Figure 3(b)). We further explored the enrichment of biological processes and pathways in this cluster. The proteins were mainly enriched in “ribosome biogenesis,” “ncRNA processing,” “rRNA processing in the nucleus and cytosol,” “snoRNA metabolic process,” and “RNA phosphodiester bond hydrolysis” (Figure 3(c)). This result was in accordance with the enrichment determined by GSEA and Proteomaps analysis. Using this approach, we not only identified and specified the core biological processes and pathways of the N-A process but also confirmed the accuracy of the data-mining analysis. Details of the primary and member enrichment are shown in Supplementary Figure 2. The enrichment annotations of each protein in MCODE1 of the N-A process are listed in Supplementary File 5.

In the A-C process, 692 upregulated DEGs were identified in GSE 117606 and 1094 upregulated DEGs in GSE 50115 by comparison of adenoma and carcinoma tissues. A total of 29 intersected genes were identified in the overlap of upregulated DEGs of the two transcriptomic datasets in the N-A process and DEPs in the A-C and N-C processes (Figure 3(d), Supplementary File 4). The PPI network and two MCODE clusters were illustrated in Figure 3(e). The seed proteins of MCODE1 and MCODE2 were IGFBP7 and CDK1, respectively. The hinge protein of the two clusters was SERPINH1. These three proteins were regarded as candidate key proteins in the A-C process. In MCODE1, the proteins were mainly enriched in “extracellular structure organization” and “posttranslational protein phosphorylation.” In MCODE2, the proteins were mainly enriched in “cell cycle, mitotic” and “activation of protein kinase activity” (Figure 3(f)). The enrichment analysis implicated ECM-related processes or pathways as one of the core events in the A-C process. The enrichment annotations of each of the proteins in MCODE1 and MCODE2 of the A-C process are listed in Supplementary File 5.

In addition to the upregulated DEPs, we also performed integrating analysis of the downregulated DEPs and DEGs in the N-A and A-C process. A total of 48 intersected genes were identified in the N-A process (Supplementary Figure 3(a)). PPI network was constructed among the 48 genes and two MCODE clusters were identified (Supplementary Figure 3(b)). The proteins in MCODE1 were mainly enriched in glycosaminoglycan metabolism and ECM-related pathways, which were different with the GSEA or Proteomaps enrichment results in the N-A process (Supplementary Figure 3(c)). The enrichment analysis of proteins in MCODE2 could not be performed because of the insufficient protein number. For the A-C process, 11 intersected genes were identified (Supplementary Figure 3(d)). PPI network could not be constructed based on these 11 proteins, and enrichment analysis was failed (Supplementary Figure 3(e)). It could be seen that the enrichment results of the downregulated DEPs were quite different from those of total DEPs, while the enrichment results of upregulated DEPs were similar to those of total DEPs. This phenomenon indicates that the upregulated DEPs are more representative in the proteomic changes in the N-A-C sequence compared with downregulated DEPs. Additionally, the upregulated protein has the advantage of being a drug target because it is more likely to be interfered by small molecules. The screening of key upregulated proteins in the N-A or A-C process could provide potential drug targets for clinical use. Hence, we mainly focused on the upregulated DEPs in the N-A-C sequence.

RRP12, SERPINH1, IGFBP7, and CDK1 were identified as candidate key proteins in the N-A-C sequence by integration analysis. We then used the “cytoHubba” plugin in Cytoscape to verify the importance of the four proteins in the whole network. cytoHubba facilitates the exploration of hub proteins in the network by ranking the proteins according to different algorithms. We selected six algorithms to determine whether the four candidate key proteins were among the top five hub proteins in their PPI networks. The four proteins were ranked in a heatmap as shown in Figure 3(g). Only RRP12 and SERPINH1 were identified consistently among the top five hub proteins in the network of the N-A and A-C processes, respectively, using all six algorithms. These two proteins were therefore regarded as key proteins in the N-A-C sequence. To validate the expression of the two proteins in the N-A-C sequence, we also analyzed the data obtained by Wisniewski et al. in a similar proteomics analysis of normal, adenoma, and carcinoma tissues [29]. This evaluation showed that RRP12 and SERPINH1 were also significantly upregulated in the N-A and A-C processes, respectively. Furthermore, there were no significant differences in the expression of RRP12 and SERPINH1 in the A-C and N-A processes, respectively (Figure 3(h)). Hence, the variation in the expression of RRP12 and SERPINH1 was verified in the N-A-C sequence (Figure 3(h)).

### 3.4. Validation of the Discrimination Power of RRP12 and SERPINH1

After identification and verification of RRP12 and SERPINH1 as key proteins in the N-A-C sequence, we validated the discrimination power of RRP12 and SERPINH1 in the N-A and A-C processes, respectively, using ROC analysis based on two GEO datasets and our own database. In the GSE 20916 dataset, for the macro dissection tissue, the AUC values of RRP12 in the N-A and N-C processes were 0.961 and 0.877, respectively (Supplementary Figure 4(a)). The AUC values of SERPINH1 in the A-C and N-C processes were 0.766 and 0.929, respectively (Supplementary Figure 4(b)). In the GSE 41567 dataset, the AUC values of RRP12 in the N-A and N-C processes were 0.967 and 1.000, respectively (Supplementary Figure 4(c)). The AUC values of SERPINH1 in the A-C and N-C processes were 0.758 and 0.967, respectively (Supplementary Figure 4(d)).

**Figure 4:**
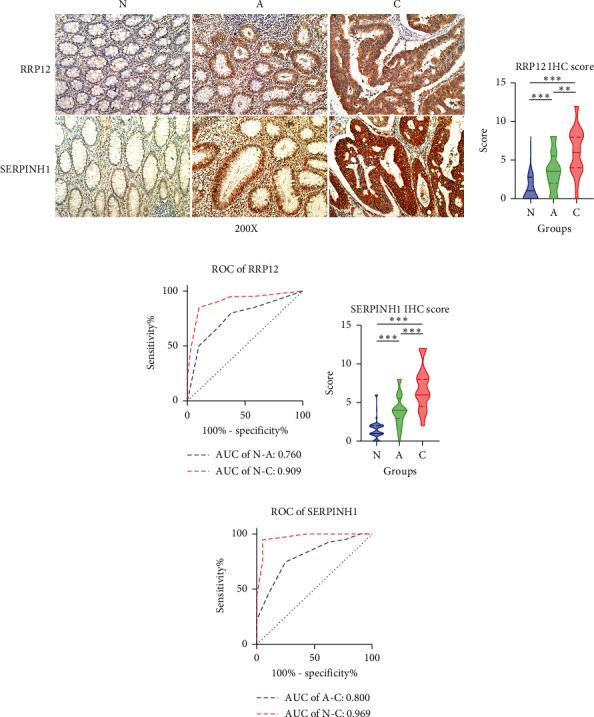
Immunohistochemical analysis to validate the discrimination power in the N-A-C sequence. (a) Representative images of RRP12 and SERPINH1 IHC staining in normal, adenoma, and carcinoma tissues. (b) In 40 paired normal, adenoma, and cancer tissues, the RRP12 expression level increased significantly in the N-A-C sequence. (c) In ROC analysis, the AUC values of RRP12 in the N-A and N-C processes were 0.760 and 0.909, respectively. (d) In 40 paired normal, adenoma, and cancer tissues, the SERPINH1 expression level increased significantly in the N-A-C sequence. (e) In ROC analysis, the AUCs of SERPINH1 in the A-C and N-C processes were 0.800 and 0.969, respectively.

We performed IHC analysis to validate the discrimination power of RRP12 and SERPINH1 based on our own database of 40 patients. To minimize the bias from different patients, we selected paired normal, adenoma, and carcinoma tissues obtained from the same patient. Representative images of RRP12 and SERPINH1 IHC staining are shown in Figure 4(a). The expression levels of both RRP12 and SERPINH1 were significantly increased in the N-A and A-C processes (Figures 4(b) and 4(d)). The AUC values of RRP12 in the N-A and N-C processes were 0.760 and 0.909, respectively (Figure 4(c)). The AUC values of SERPINH1 in the A-C and N-C processes were 0.800 and 0.969, respectively (Figure 4(e)).

### 3.5. RRP12 and SERPINH1 Knockdown Reduces the Viability and Proliferation of Adenoma Cells in Organoids

**Figure 5:**
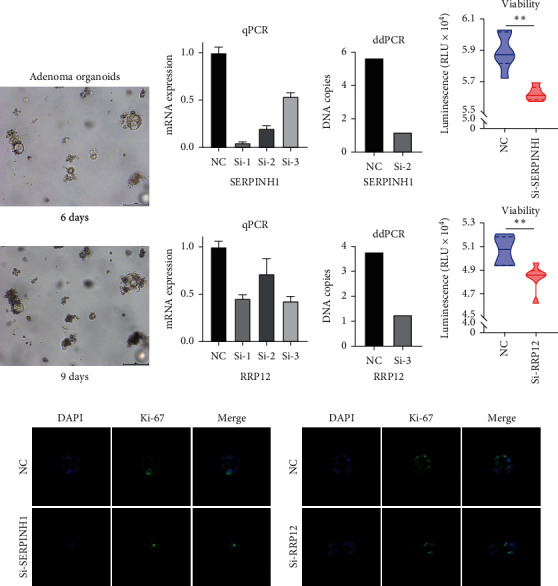
RRP12 and SERPINH1 knockdown reduces viability and proliferation of adenoma organoids, respectively. (a) Representative images of adenoma organoids after 6 and 9 days of culture. (b) The mRNA expression levels of SERPINH1 and RRP12 in SW480 cells were evaluated by qPCR after knockdown mediated by 3 siRNA sequences. (c) SERPINH1 and RRP12 DNA copy number in SW480 cells was evaluated by ddPCR after knockdown mediated by siRNA sequences 2 and 3, respectively. (d) The viability of adenoma organoids significantly decreased after SERPINH1 and RRP12 knockdown compared with the control group. (e) Ki-67 immunostaining showed decreased proliferation of adenoma organoids after SERPINH1 and RRP12 knockdown compared with the control group.

We speculated that RRP12 and SERPINH1 functions act as key proteins in the N-A and A-C processes based on the good discriminatory ability of these proteins and their pivotal roles in the core biological processes. Viability is a significant characteristic of epithelial cells in the N-A and A-C processes during carcinogenesis. Therefore, we explored the influence of RRP12 or SERPINH1 on the viability of adenoma cells at the organoid level. Representative images of adenoma organoids after 6 and 9 days of culture are shown in Figure 5(a). The expression of RRP12 and SERPINH1 was knocked down in SW480 cells by transfection with specific siRNAs (Figure 5(b)). We then used this approach to achieve knockdown of RRP12 and SERPINH1 in adenoma organoids established from fresh surgical specimens; the effectiveness of the knockdown process was confirmed by ddPCR (Figure 5(c)). After knockdown of RRP12 or SERPINH1, adenoma organoids showed decreased cell viability and fewer Ki67-positive cells, indicating a reduction in adenoma cell proliferation (Figures 5(d)–5(e)). These results indicated that RRP12 and SERPINH1 play crucial roles in the N-A and A-C processes.

### 3.6. SERPINH1 Is a Risk Factor for DFS and Correlates with Increased TNM Stages and EMT Phenotype

**Figure 6:**
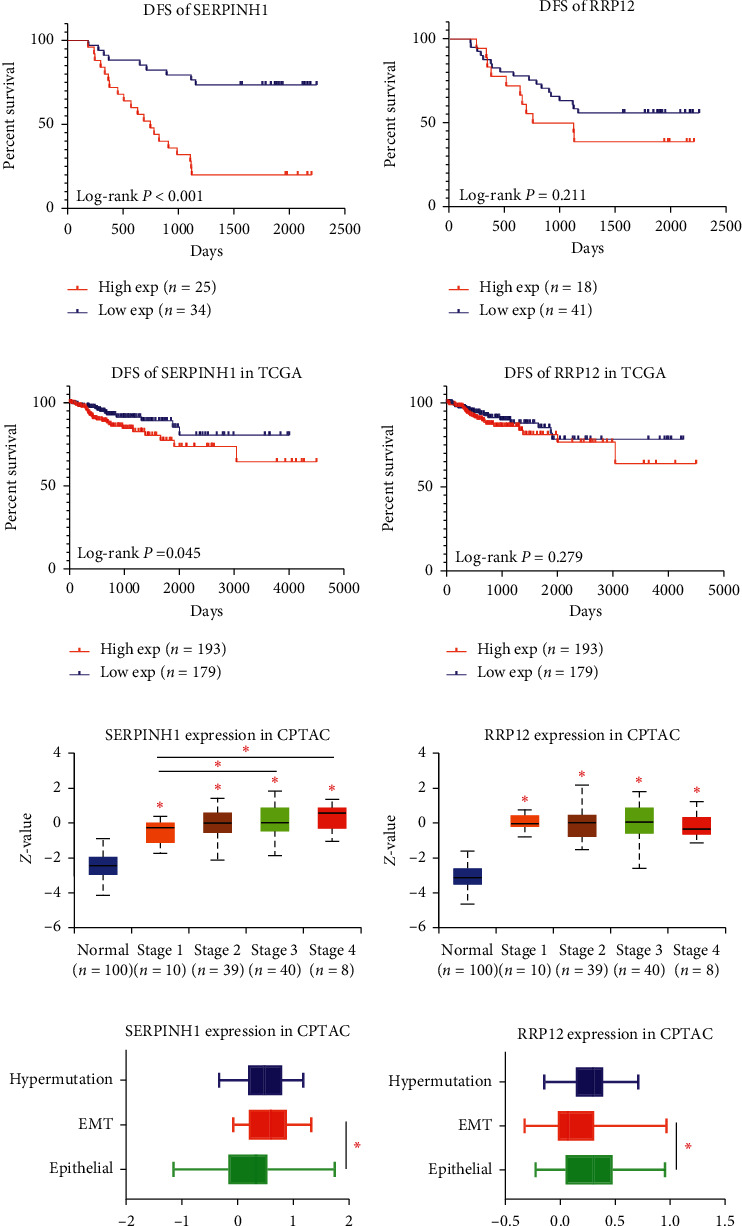
SERPINH1 is a risk factor for DFS and correlates with increased TNM stage and EMT phenotype. (a) The DFS of the high SERPINH1 expression group is significantly poorer than that of the low expression group in our database (*P* < 0.001, 59 patients). (b) There is no significant difference in the DFS of the high RRP12 expression group (IHC score ≥9 points) compared with the low expression group in our database (IHC score <9 points) (*P*=0.211, 59 patients). (c) The DFS of the high SERPINH1 expression group is significantly poorer than that of the low expression group in the TCGA database (*P*=0.045, 372 patients). (d) There is no significant difference in the DFS of the high RRP12 expression group compared with the low expression group in the TCGA database in DFS (*P*=0.279, 372 patients). (e) In the CPTAC database, the expression level of SERPINH1 is significantly higher in the stage I–IV groups compared with the normal group. Furthermore, the SERPINH1 expression level is higher in the stage III and stage IV groups compared with that in the stage I group (data obtained from UALCAN). (f) In the CPTAC database, the RRP12 expression level is significantly higher in the stage I–IV groups compared with that in the normal group (data obtained from UALCAN). (g) In the CPTAC database, the SERPINH1 expression level in the EMT group is significantly higher than that in the epithelial group (data obtained from cBioPortal). (h) In the CPTAC database, the RRP12 expression level in the EMT group is significantly lower than that in the epithelial group (data obtained from cBioPortal).

After validating the discriminatory power and biological behavior of RRP12 and SERPINH1, we explored the potential of RRP12 or SERPINH1 as prognostic markers. We performed survival analysis based on IHC score for our database of 59 colorectal cancer patients. The high SERPINH1 IHC score (≥9 points) group showed significantly decreased DFS compared with the low SERPINH1 IHC score (<9 points) group (*P* < 0.001, Figure 6(a)). However, there was no significant difference in the DFS of the high and low RRP12 IHC score groups (*P*=0.211, Figure 6(b)). We then combined the clinicopathological factors with the IHC scores for RRP12 or SERPINH1 in these patients to perform univariate and multivariate analysis (Table 1). In the multivariate analysis, SERPINH1 was identified as an independent prognostic factor for DFS. Then we used TCGA database to validate the prognostic value of SERPINH1 and RRP12. The high SERPINH1expression group showed significantly decreased DFS compared with that in the low expression group (*P*=0.045, Figure 6(c)). In contrast, RRP12 did not show prognostic ability in TCGA database (*P*=0.279, Figure 6(d)).

The CPTAC database combined quantitative proteomic data and clinicopathological information. Therefore, we explored the correlations between the two proteins and TNM stage. RRP12 and SERPINH1 expression levels in the different TNM stages were significantly higher than those in normal tissues (Figures 6(e)–6(f)). Furthermore, SERPINH1 expression was significantly higher in the stage III and stage IV patients compared with that in the stage I patients (Figure 6(e)). In contrast, there were no significant differences in RRP12 expression levels among the different TNM stages (Figure 6(f)). According to this analysis, we speculated that the favorable prognostic value of SERPINH1 is based on the key involvement of this protein in the A-C process (carcinogenesis) as well as the progression (local and distant metastasis) of cancer. In the functional analysis of the A-C process, the proteomic variation was found to be enriched in EMT- and ECM-related pathways. One piece of the annotation information of SERPINH1 was annotated in EMT in the Hallmark gene set (Supplementary File 5). Moreover, EMT is one of the most crucial processes in the metastasis of colorectal cancer. Therefore, we explored the correlation between SERPINH1 expression levels and the EMT phenotype based on the CPTAC database. The expression level of SERPINH1 was shown to be significantly higher in the EMT group compared with that in the epithelial group (Figure 6(g)). In contrast, the expression level of RRP12 was significantly higher in the epithelial group than that in the EMT group (Figure 6(h)).

### 3.7. SERPINH1 Correlates with EMT and Promotes Cancer Development In Vitro

**Figure 7:**
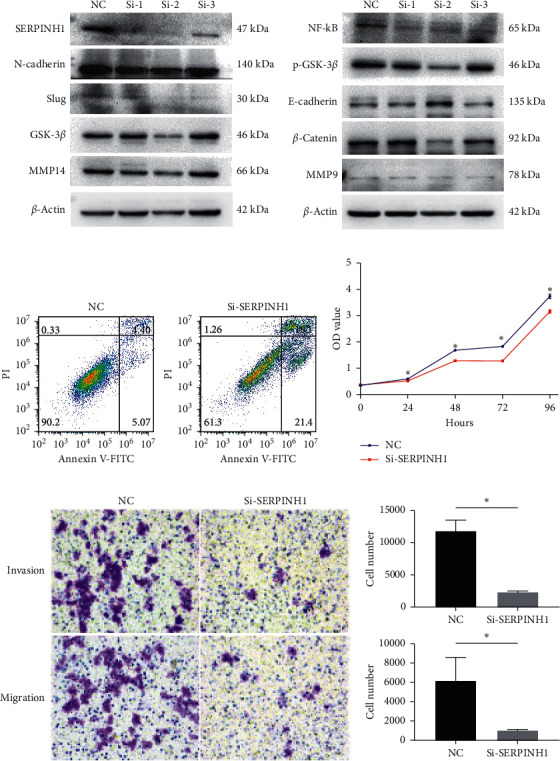
SERPINH1 correlates with EMT and promotes cancer development in vitro. (a) Western blot analysis showing that SERPINH1 siRNA sequence 2 mediated the best knockdown effect. In SW480 cells with SERPINH1 knockdown mediated by siRNA sequence 2, E-cadherin expression is increased compared with that in the controls, while the expressions of N-cadherin, *β*-catenin, Slug, GSK-3*β*, p-GSK-3*β*, NF-kB, MMP9, and MMP14 are reduced. There are no significant variations in the expressions of EMT-related proteins in SW480 cells with SERPINH1 knockdown mediated by siRNA sequences 1 and 3. (b) Flow cytometric analysis showing a higher rate of apoptosis in the si-SERPINH1 knockdown SW480 cells compared with that in the control group. (c) Viability analysis showing reduced proliferation ability in the si-SERPINH1 knockdown SW480 cells compared with that in the control group. (d) Transwell analysis showing decreased invasion and migration ability in the si-SERPINH1 knockdown SW480 cells compared with that in the control group. (e) Histogram plots showing significantly decreased numbers of invading (upper plot) and migrating (lower plot) cells in the si-SERPINH1 knockdown SW480 cells compared with those in the control group.

Based on these results, we hypothesized that SERPINH1 correlates positively with EMT and metastasis. To validate the relationship between SERPINH1 and EMT, we performed Western blot analysis to evaluate the variation in the expression of crucial EMT-related proteins after SERPINH1 knockdown. Three siRNA sequences were evaluated for their ability to mediate SERPINH1 knockdown in SW480 cells; and siRNA sequence 2 showed the best knockdown effect (Figure 7(a)). After transfection with this siRNA, the protein levels of N-cadherin, *β*-catenin, Slug, GSK-3*β*, p-GSK-3*β*, NF-kB, MMP9, and MMP14 were downregulated, while E-cadherin expression was upregulated. In contrast, there were no significant differences in the expression levels of EMT-related proteins after transfection with SERPINH1-specific siRNA sequences 1 and 3 (Figure 7(a)). Based on these observations, we chose the siRNA sequence for use in the subsequent functional experiments and speculated that SERPINH1 may correlate with EMT.

To explore the potential of SERPINH1 to promote cancer development, we analyzed the effect of SERPINH1 knockdown on SW480 cell apoptosis. After transfection with SERPINH1-specific siRNA sequence for 48 h, flow cytometric analysis of Annexin-V staining revealed increased cell death in the SERPINH1 knockdown cells (Figure 7(b)). CCK8 assays also showed reduced proliferation of SW480 cells after SERPINH1 knockdown (Figure 7(c)). Furthermore, Transwell assays revealed decreased invasion and migration ability of SW480 cells after SERPINH1 knockdown (Figures 7(d)–7(e)). These results indicated that SERPINH1 reduces apoptosis, while promoting the proliferation, invasion, and migration of CRC cells. Based on these results, we speculated that SERPINH1 correlates with EMT and promotes the development of cancer.

## 4. Discussion

The process of carcinogenesis in CRC can be defined by a typical model involving sequential phases of changes from normal tissue to a premalignant lesion and progression to local carcinoma. Thirty years ago, in accordance with the mutation accumulation theory formulated by Nordling, Vogelstein and Fearon proposed the canonical genetic model of the N-A-C sequence in CRC [30, 31]. Although the model was confirmed and is widely accepted, some issues remained unresolved until recently. In the N-A-C sequence, the *APC* mutation is the core incident of adenoma formation in both humans and mouse models [32]. Lahouel et al. explained this phenomenon by categorizing the mutations into three types (cell fate, cell survival, and genome maintenance) and established a model combining epidemiologic and sequencing data [33]. Tomasetti et al. found that only three driver mutations are required to convert the normal mucosa to cancer and this conclusion changed the conventional concept that carcinogenesis requires at least six mutations [34]. This phenomenon indicates that although the course of the N-A-C sequence is prolonged, the pivotal gene mutation events might not be complicated. With the development of omics technologies, an increasing number of studies have elucidated the pivotal events of the N-A-C sequence at different levels through genomics, epigenomics, transcriptomics, proteomics, metabolomics, and microbiomics [6, 13, 35–37]. In this study, we aimed to identify key proteins that play an important role in the N-A-C sequence through in-depth proteomics analysis following the “identification-verification-validation” procedure.

In the identification phase, we initially marked differences in the proteomic variations between the N-A and A-C processes, indicating that the progression occurs in two phases with differences in protein expression. Therefore, we conducted a bioinformatic analysis of the N-A and A-C processes. We integrated the proteomic profiles and external transcriptomic datasets, constructed PPI networks, extracted the MCODE clusters, and identified candidate key proteins (RRP12, SERPINH1, IGFBP7, and CDK1) for the N-A and A-C processes. In the verification step, we confirmed RRP12 and SERPINH1 as the key proteins in the N-A and A-C processes, respectively, by using cytoHubba to rank hub proteins and an external proteomic database to evaluate the expression level. In the validation phase, we confirmed the discrimination value of RRP 12 and SERPINH1 in our own dataset and databases. Furthermore, the two proteins were positively correlated with the viability and proliferation of adenoma organoids. SERPINH1 was identified as a risk factor for DFS in TCGA and our own database. Finally, based on the results of Western blot and functional studies, we speculated that SERPINH1 may correlate with EMT and promote the development of cancer. Thus, our study elucidates the proteomic variation enrichment in the N-A and A-C processes and clarifies the evolution of the N-A-C sequence in protein level. Furthermore, the majority of previous studies designed to identify biomarkers of the N-A-C sequence were focused on the discrimination value. In the current study, a key protein, SERPINH1, in the A-C process was screened out, and it was found to be of discriminatory and prognostic value. Therefore, SERPINH1 may become a candidate biomarker and a potential therapeutic target for clinical application. These findings were further supported by the results of our functional studies on both and cancer cell lines adenoma organoids.

We found that ribosome- and spliceosome-related events were significantly enriched in the N-A process. Ribosomes are important organelles for protein synthesis and are composed of ribosomal proteins (RPs) and RNAs (rRNAs). Although the main function of RPs is structural, the important role of RPs in tumorigenesis cannot be ignored. Leonart et al. reported that some RPs are associated with oncogene activation, and dysfunction of RPs may contribute to tumorigenesis directly [38]. Xu and Lai proposed that the perturbation and extraribosomal functions of RPs play an important role in the tumorigenesis of CRC [39]. RRP12 is a nucleolar protein that participates in synthesis and nuclear export of 40S ribosomal subunits [40]. It has been reported that RRP12 is regulated by hsa-miR-140-3p and hsa-miR-200c in basal II breast cancer and may play an important role of tumor differentiation [41]. POLR1B regulates RRP12 to promote proliferation of non-small-cell lung cancer [42]. In an osteosarcoma cell line, RRP12 was shown to negatively regulate TP53 expression and was implicated as a target to improve the effect of chemotherapy [43]. Pre-mRNA splicing is a pivotal process in the formation of mature mRNA and regulation of gene expression. Aberrant splicing occurs in multiple types of cancer and targeting the splicing machinery has been highlighted as a novel anticancer strategy [44]. Some specific isoforms produced by aberrant splicing (mRNA and/or protein) can promote tumor progression and genomic instability [45, 46]. RNA splicing factors can also be regarded as oncosuppressor or tumor-suppressor proteins [47].

We found that ECM- and EMT-related pathways were significantly enriched in the A-C process. The ECM is a crucial structure in the tumor microenvironment. Under normal conditions, the primary function of the ECM is to maintain homeostasis and orchestrate tissue repair in response to injury or damage [48]. In the ECM, the basement membrane consists mainly of collagen, integrins, laminin, and proteoglycans and forms an inherent barrier against tumor invasion and metastasis [49]. The basement membrane can be destroyed by protease-dependent chemicals and/or force-driven physical mechanisms [49]. Chaudhuri and Nam showed that the process of cell division generates significant protrusive force that deforms the surroundings along the mitotic axis in a three-dimensional model [50]. This discovery not only presents a novel physical perspective of invasion but also clarifies the relationship between cell proliferation and invasion. Moreover, in our study, the proteomic variation landscape of the N-A-C sequence was found to be somewhat consistent with the chronology of proliferation and invasion. Ribosomal and spliceosome proteins related to proliferation were mainly enriched in the N-A process. In the A-C process, the invasion capability of neoplastic cells is enhanced and the damaged ECM is reconstructed. SERPINH1 binds specifically to collagen and functions as a chaperone in the biosynthetic pathway. In breast cancer, SERPINH1 is a hub gene of ECM transcription network and promotes tumor growth [51]. In a very recent study, SERPINH1 was shown to induce cancer cell-platelet interaction through type I collagen and promote cancer metastasis [52]. Therefore, the SERPINH1-collagen axis is a promising therapeutic target [52]. In CRC, Mori et al. found that SERPINH1 is a predictive biomarker of lymph node metastasis rather than distant metastasis [53]. It is widely accepted that EMT contributes to cancer development and metastasis. In this process, epithelial cells lose their characteristically tight conjunction and polarity and develop the phenotype and invasion/migration capacity of mesenchymal cells. In our study, we validated the prognostic value of SERPINH1 and identified a potential correlation with EMT. Furthermore, our functional studies showed that the invasion and migration ability was reduced in SW480 cells following SERPINH1 knockdown. Thus, we speculate that SERPINH1 plays a key role in both carcinogenesis and cancer development.

Some limitations of this study should be noted. First, the results are mainly based on proteomic analysis and functional studies, and the potential mechanisms underlying the roles of RRP12 and SERPINH1 in the N-A-C sequence remain to be elucidated. An in-depth exploration of the mechanism by which SERPINH1 promotes cancer development is also warranted. Second, our analysis was performed on a small number of clinical samples and the prognostic ability of the candidate key proteins identified in this study requires validation in a large cohort. Third, this study was based on only proteomic and transcriptomic analyses and the events that drive the N-A-C sequence occur at the genetic level. Our analysis showed that metabolism-related pathways were obviously enriched in the A-C process. Therefore, a combination of genomics and metabolomics approaches may provide a more comprehensive understanding of the N-A-C sequence.

There are two significances of this study: (1) This study indicated that RRP12 and SERPINH1 were the key proteins in the N-A process and the A-C process, respectively. This finding would contribute to target prevention of the carcinogenesis in CRC and then accelerate secondary prevention of CRC precisely. (2) SERPINH1 might play a key role in both the A-C process and the development of CRC. Moreover, SERPINH1 could be secreted into extracellular space. Hence, SERPINH1 could potentially be the noninvasive biomarker reflecting the adenoma and carcinoma progression.

## 5. Conclusion

In conclusion, we comprehensively analyzed the proteomic variations in the N-A-C sequence. Ribosome- and spliceosome-related pathways were mainly enriched in the N-A process, whereas ECM- and EMT-related pathways were mainly enriched in the A-C process. RRP12 and SERPINH1 may play an important role in the N-A and A-C processes, respectively. Furthermore, SERPINH1 showed favorable prognostic value for DFS in CRC patients. We speculate that SERPINH1 might promote not only the A-C process but also the development of CRC.

## Figures and Tables

**Table 1:** Univariate and multivariate analysis of 59 CRC patients.

Variables	Univariate analysis			Multivariate analysis		
	HR	95% CI	*P*	HR	95% CI	*P*
Sex						
Female	Reference					
Male	1.023	0.494–2.120	0.951			

Age						
<65	Reference			Reference		
≥65	2.084	0.948–4.583	**0.068**	1.554	0.666–3.626	0.308

Histological subtype						
Adenocarcinoma	Reference			Reference		
MUC + SRCC	4.069	1.784–9.281	**0.001**	2.819	0.941–8.444	0.064

T stage						
1 + 2	Reference					
3	0.998	0.363–2.479	0.997			
4	2.172	0.725–6.508	0.166			

N status						
−	Reference			Reference		
+	1.974	0.917–4.253	**0.082**	1.811	0.784–4.179	0.164

Tumor location						
Right	Reference			Reference		
Left	0.409	0.166–1.007	**0.052**	0.415	0.147–1.167	0.095

VI						
−	Reference			Reference		
+	7.051	3.306–15.037	**<0.001**	2.076	0.734–5.866	0.168

RRP12 expression						
Low	Reference					
High	1.609	0.759–3.414	0.215			

SERPINH1 expression						
Low	Reference			Reference		
High	4.806	2.161–10.091	**<0.001**	3.086	1.148–8.297	**0.026**

HR: hazard ratio; 95% CI: 95% confidence interval; MUC: mucinous adenocarcinoma; SRCC: signet ring cell carcinoma; VI: vascular invasion.

## Data Availability

All data generated or analyzed during this study are included in the supplementary files. The mass spectrometry proteomics data have been deposited to the ProteomeXchange Consortium (http://proteomecentral.proteomexchange.org) via the iProX partner repository with the dataset identifier: PXD017068 and PXD023899.

## Supplementary Materials

Supplementary Materials Supplementary File 1: quantitative analysis of the identified proteins in the first replicate. Supplementary File 2: quantitative analysis of the identified proteins in the second replicate. Supplementary File 3: quantitative analysis of the identified proteins in the third replicate. Supplementary File 4: the intersected proteins in the N-A and A-C processes. Supplementary File 5: the enrichment annotation of each protein of the MCODE clusters in the N-A-C sequence. Table S1: clinical and pathological characteristics of the patients. Supplementary Figure 1: workflow of this study. Supplementary Figure 2: the primary and member biological process and pathway enrichment of MCODE1 in the N-A process. Supplementary Figure 3: integrating analysis of downregulated DEPs in the N-A and A-C processes. Supplementary Figure 4: ROC curves of RRP12 and SERPINH1 based on GEO database.
