# Supplementary material for: Exploration of the Key Proteins in the Normal-Adenoma-Carcinoma Sequence of Colorectal Cancer Evolution Using In-Depth Quantitative Proteomics
Source: J Oncol. 2021 Jun 11;2021:5570058. doi: 10.1155/2021/5570058 (PMC8214504; doi:10.1155/2021/5570058)
Supplement: Supplementary Materials — Supplementary File 1: quantitative analysis of the identified proteins in the first replicate. Supplementary File 2: quantitative analysis of the identified proteins in the second replicate. Supplementary File 3: quantitative analysis of the identified proteins in the third replicate. Supplementary File 4: the intersected proteins in the N-A and A-C processes. Supplementary File 5: the enrichment annotation of each protein of the MCODE clusters in the N-A-C sequence. Table S1: clinical and pathological characteristics of the patients. Supplementary Figure 1: workflow of this study. Supplementary Figure 2: the primary and member biological process and pathway enrichment of MCODE1 in the N-A process. Supplementary Figure 3: integrating analysis of downregulated DEPs in the N-A and A-C processes. Supplementary Figure 4: ROC curves of RRP12 and SERPINH1 based on GEO database. [file 5570058.f1.zip › Supplementary Figures & tables.docx]

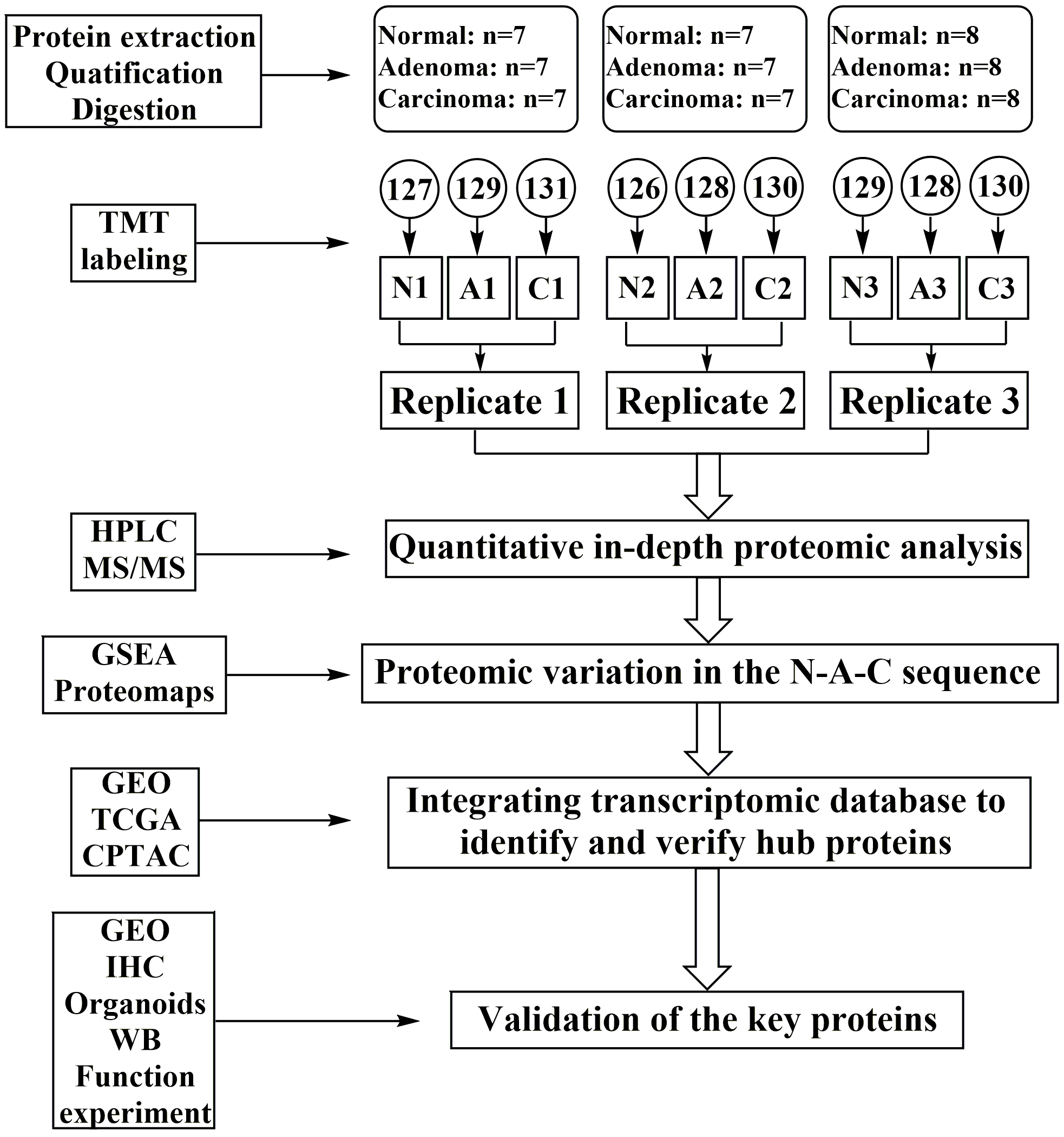


**Supplementary Figure 1 Workflow of this study**

Replicate 1: The first replicate; Replicate 2: the second replicate; Replicate 3: the third replicate.


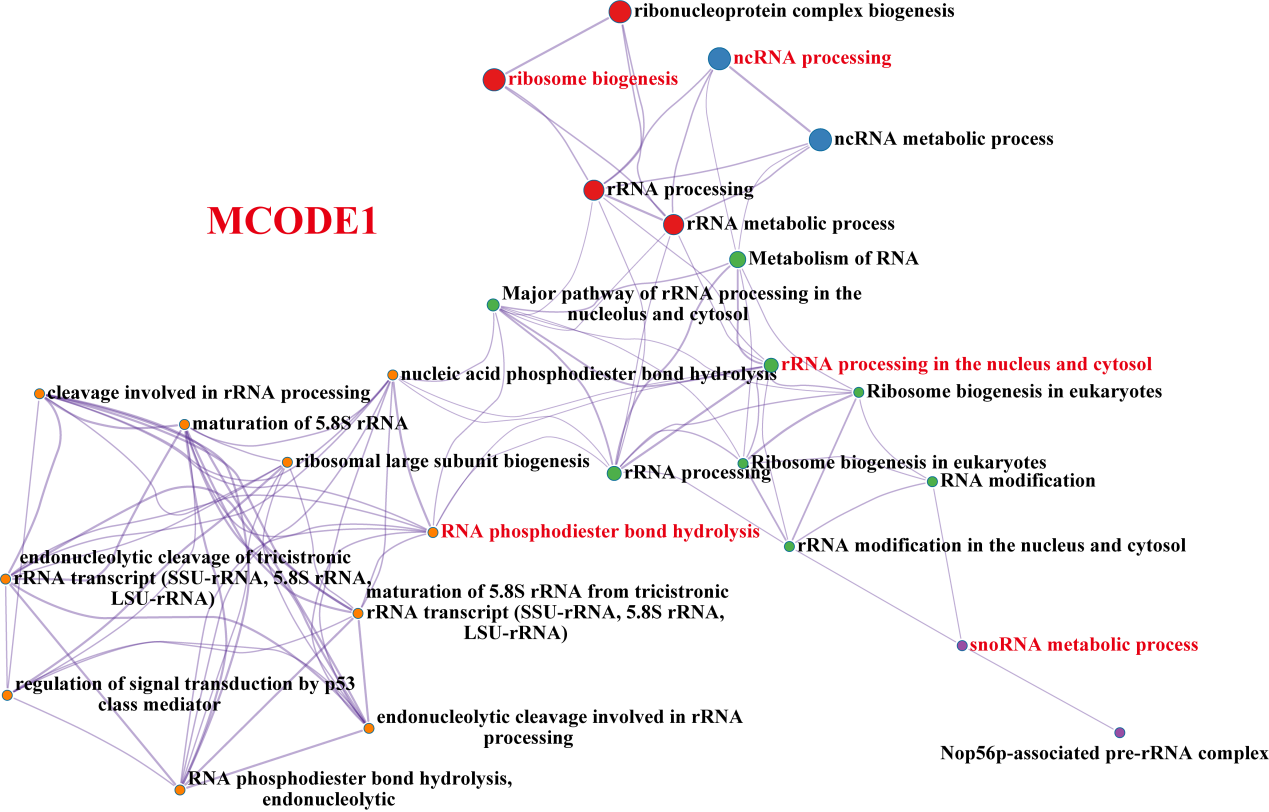


**Supplementary Figure 2 The primary and member biological process and pathway enrichment of MCODE1 in the N-A process.**

The primary and member biological process and pathway enrichment of MCODE1 in the N-A process. The primary enrichment results are marked in red font and the node color of the member enrichment results are in accordance with corresponding enrichment result. The size of each node reflects the number of proteins enriched in this biological process or pathway.

**
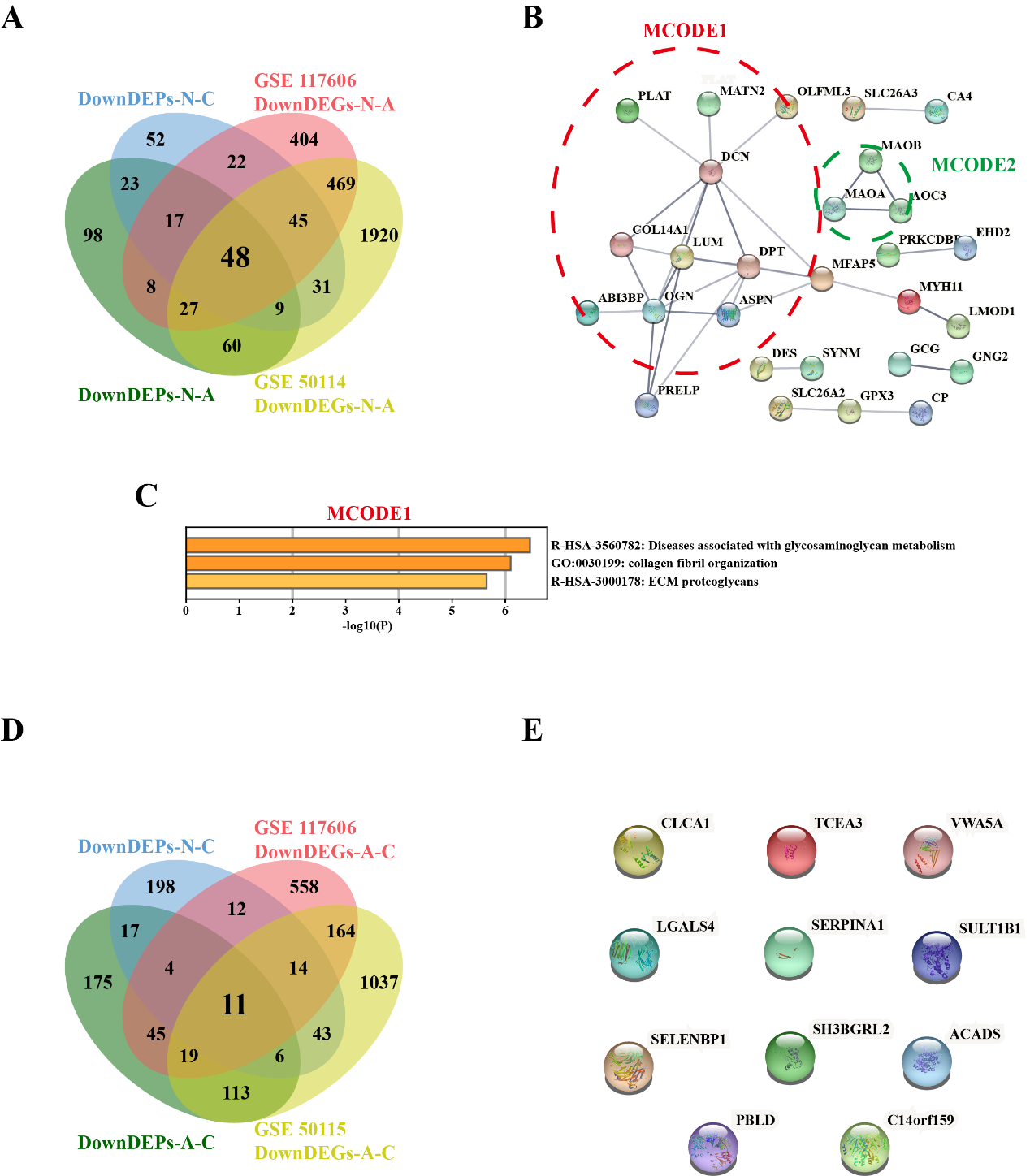
**

**Supplementary Figure 3** **Integrating analysis of downregulated DEPs in N-A and A-C processes.**

(a) Venn diagram showing the intersection of downregulated DEPs in the N-C process (DownDEPs-N-C), downregulated DEPs in the N-A process (DownDEPs-N-A), and downregulated DEGs of GSE 117606 and GSE 50114 in the N-A process. A total of 48 intersected genes in the four datasets were identified. (b) The PPI network and MCODE analysis of the 48 intersected proteins. (c) Enrichment analysis of MCODE1. (d) Venn diagram showing the intersection of DownDEPs-N-C, downregulated DEPs in the A-C process (DownDEPs-A -C), and downregulated DEGs of GSE 117606 and GSE 50115 in the A-C process. A total of 11 intersected genes in the four datasets were identified. (e) The 11 proteins failed to construct PPI network.

**
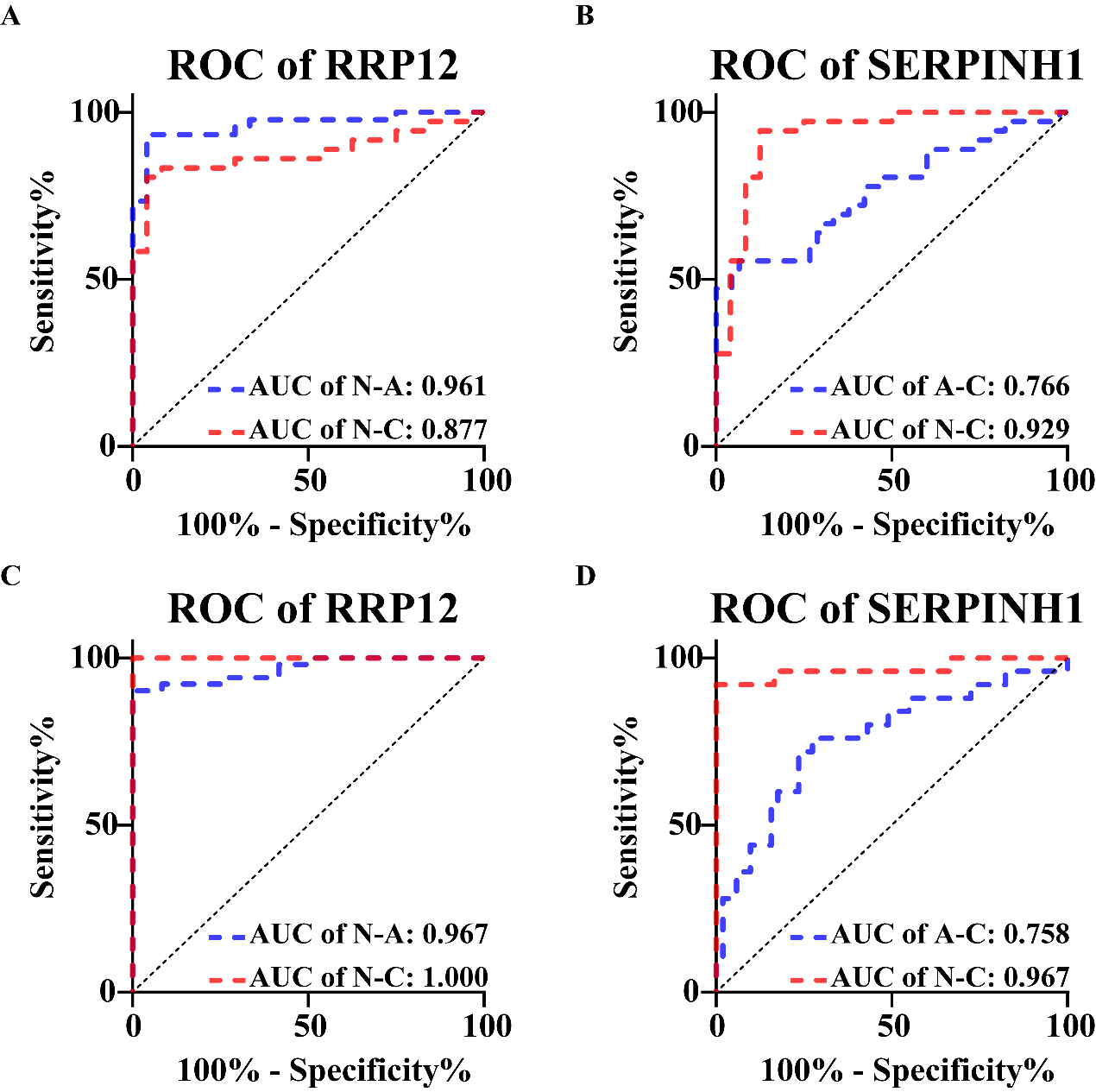
**

**Supplementary Figure 4 ROC curves of RRP12 and SERPINH1 based on GEO database.**

(a) ROC curves of RRP12 in the N-A and N-C processes based on GSE 21906 (macro dissection). (b) ROC curves of SERPINH1 in the A-C and N-C processes based on GSE 21906 (macro dissection). (c) ROC curves of RRP12 in the N-A and N-C processes based on GSE 41567. (d) ROC curves of SERPINH1 in the A-C and N-C processes based on GSE 41567.

**Table S1. Clinical and pathological characteristics of the patients**

| Replicate 1&2 | | | | | | |  | Replicate 3 | | | | | | |
| --- | --- | --- | --- | --- | --- | --- | --- | --- | --- | --- | --- | --- | --- | --- |
| Patient | Sex | Age | Pathology | Location | Stage | Differentiation |  | Patient | Sex | Age | Pathology | Location | Stage | Differentiation |
| 1 | M | 59 | N | Sc | - | - |  | 8 | M | 57 | N | Asc | - | - |
| 2 | M | 72 | N | R | - | - |  | 9 | F | 50 | N | Asc | - | - |
| 3 | F | 64 | N | Asc | - | - |  | 10 | F | 68 | N | R | - | - |
| 4 | M | 56 | N | R | - | - |  | 11 | M | 59 | N | Asc | - | - |
| 5 | F | 59 | N | Sc | - | - |  | 12 | M | 73 | N | R | - | - |
| 6 | M | 65 | N | R | - | - |  | 13 | F | 54 | N | Sc | - | - |
| 7 | M | 62 | N | Asc | - | - |  | 14 | F | 68 | N | Sc | - | - |
|  |  |  |  |  |  |  |  | 15 | M | 80 | N | Asc | - | - |
| 1 | M | 59 | TA | R | - | - |  | 16 | M | 75 | TA | Sc | - | - |
| 2 | M | 72 | TA | R | - | - |  | 17 | F | 64 | TA | R | - | - |
| 3 | F | 64 | TA | Asc | - | - |  | 18 | F | 47 | TA | Tc | - | - |
| 4 | M | 56 | TA | Ce | - | - |  | 19 | F | 68 | TA | RSc | - | - |
| 5 | F | 59 | TVA | Ce | - | - |  | 20 | M | 58 | TA | Dsc | - | - |
| 6 | M | 65 | TVA | R | - | - |  | 21 | M | 55 | TA | Asc | - | - |
| 7 | M | 62 | TA | Asc | - | - |  | 22 | F | 63 | TA | R | - | - |
|  |  |  |  |  |  |  |  | 23 | M | 67 | TA | Hf | - | - |
| 1 | M | 59 | AC | Sc | T3N0M0 | Moderate |  | 8 | M | 57 | AC | Ce | T3N0M0 | Moderate |
| 2 | M | 72 | AC | R | T3N1aM0 | Well-Moderate |  | 9 | F | 50 | AC | Ce | T3N0M0 | Moderate |
| 3 | F | 64 | AC | Ce | T3N1bM0 | Moderate |  | 10 | F | 68 | AC | R | T2N0M0 | Well-Moderate |
| 4 | M | 56 | AC | R | T3N1cM0 | Moderate |  | 11 | M | 59 | AC | Ce | T3N1bM0 | Well-Moderate |
| 5 | F | 59 | AC | Sc | T3N0M0 | Moderate |  | 12 | M | 73 | AC | R | T2N2aM0 | Moderate |
| 6 | M | 65 | AC+MAC | R | T3N0M0 | Moderate |  | 13 | F | 54 | AC | Sc | T3N0M0 | Moderate |
| 7 | M | 62 | AC+MAC | Ce | T2N2bM0 | Poor |  | 14 | F | 68 | AC | Sc | T3N2bM0 | Moderate |
|  |  |  |  |  |  |  |  | 15 | M | 80 | AC | Asc | T3N0M0 | Moderate |

Abbreviations: M, male; F, female; N, normal tissue; TA, tubular adenoma; TVA, tubulovillous adenoma; AC, adenocarcinoma; MAC, mucinous adenocarcinoma; Ce, Cecum; Asc, ascending colon; Hf, hepatic flexure; Tc, transverse colon; Dsc, descending colon; Sc, sigmoid colon; Rsc, rectosigmoid colon; R, rectum.
